# (*Z*)-4-Amino-1,2,5-oxadiazole-3-carboxamide oxime

**DOI:** 10.1107/S1600536809044432

**Published:** 2009-10-31

**Authors:** Hui Zhang, Fang-fang Jian

**Affiliations:** a MicroScale Science Institute, Department of Chemistry and Chemical Engineering, Weifang University, Weifang 261061, People’s Republic of China; b MicroScale Science Institute, Weifang University, Weifang 261061, People’s Republic of China

## Abstract

The asymmetric unit of the title compound, C_3_H_5_N_5_O_2_, contains three crystallograpically independent mol­ecules. In the crystal structure, inter­molecular N—H⋯N, N—H⋯O, O—H⋯N and O—H⋯O hydrogen bonds link the mol­ecules into a three-dimensional network.

## Related literature

For background to the biological activity of 1,2,5-oxadiazo­les, see: Renaud & Sebastian (2003 ▶).
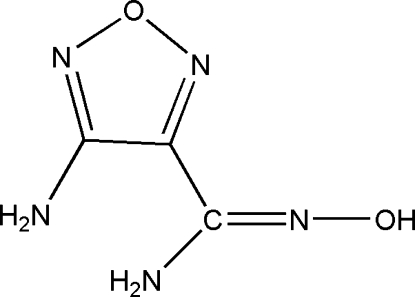


## Experimental

### 

#### Crystal data

C_3_H_5_N_5_O_2_
*M* *_r_* = 143.12
Monoclinic, 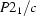

*a* = 7.6514 (15) Å
*b* = 11.712 (2) Å
*c* = 19.218 (4) Å
β = 96.53 (3)°
*V* = 1710.9 (6) Å^3^
*Z* = 12
Mo *K*α radiation
μ = 0.14 mm^−1^
*T* = 293 K
0.20 × 0.15 × 0.10 mm

#### Data collection

Bruker SMART CCD diffractometer
Absorption correction: none
16421 measured reflections
3891 independent reflections
2954 reflections with *I* > 2σ(*I*)
*R*_int_ = 0.018

#### Refinement

*R*[*F* ^2^ > 2σ(*F* ^2^)] = 0.041
*wR*(*F* ^2^) = 0.131
*S* = 0.88
3891 reflections
271 parameters
H-atom parameters constrained
Δρ_max_ = 0.41 e Å^−3^
Δρ_min_ = −0.27 e Å^−3^

### 

Data collection: *SMART* (Bruker, 1997 ▶); cell refinement: *SAINT* (Bruker, 1997 ▶); data reduction: *SAINT*; program(s) used to solve structure: *SHELXS97* (Sheldrick, 2008 ▶); program(s) used to refine structure: *SHELXL97* (Sheldrick, 2008 ▶); molecular graphics: *SHELXTL* (Sheldrick, 2008 ▶); software used to prepare material for publication: *SHELXTL*.

## Supplementary Material

Crystal structure: contains datablocks global, I. DOI: 10.1107/S1600536809044432/lh2924sup1.cif

Structure factors: contains datablocks I. DOI: 10.1107/S1600536809044432/lh2924Isup2.hkl

Additional supplementary materials:  crystallographic information; 3D view; checkCIF report

## Figures and Tables

**Table 1:** Hydrogen-bond geometry (Å, °)

*D*—H⋯*A*	*D*—H	H⋯*A*	*D*⋯*A*	*D*—H⋯*A*
O2*C*—H2*CA*⋯O2*A*	0.82	2.01	2.8222 (14)	169
O2*A*—H2⋯N4*B*	0.82	2.14	2.9208 (14)	160
N5*C*—H5*CA*⋯N3*C*^i^	0.86	2.24	3.0610 (17)	160
N5*C*—H5*CB*⋯N2*C*^ii^	0.86	2.33	3.1508 (17)	160
N1*C*—H1*CA*⋯O2*C*^iii^	0.86	2.58	3.3239 (15)	145
N1*C*—H1*CB*⋯N1*A*^iii^	0.86	2.49	3.1504 (17)	135
N1*C*—H1*CB*⋯N4*C*	0.86	2.39	2.9600 (16)	124
N5*A*—H5*AA*⋯N3*A*^iii^	0.86	2.22	3.0486 (17)	162
N5*A*—H5*AB*⋯N2*B*^iv^	0.86	2.31	3.1409 (17)	162
N1*A*—H1*AA*⋯N1*B*^i^	0.86	2.60	3.2156 (17)	130
N1*A*—H1*AB*⋯O2*B*	0.86	2.56	3.3430 (17)	153
N1*A*—H1*AB*⋯N4*A*	0.86	2.29	2.8563 (15)	123
O2*B*—H2*BA*⋯N1*C*^v^	0.82	2.07	2.8849 (15)	171
N1*B*—H1*BB*⋯N4*B*	0.86	2.34	2.8992 (15)	123
N1*B*—H1*BB*⋯N4*C*^v^	0.86	2.54	3.1510 (17)	129
N5*B*—H5*BA*⋯N3*B*^i^	0.86	2.23	3.0470 (16)	159
N5*B*—H5*BB*⋯N2*A*^vi^	0.86	2.35	3.1779 (17)	162

Symmetry codes: (i) 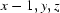; (ii) 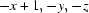; (iii) 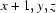; (iv) 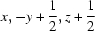; (v) 
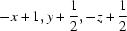
; (vi) 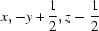.

**Fig. 1.**
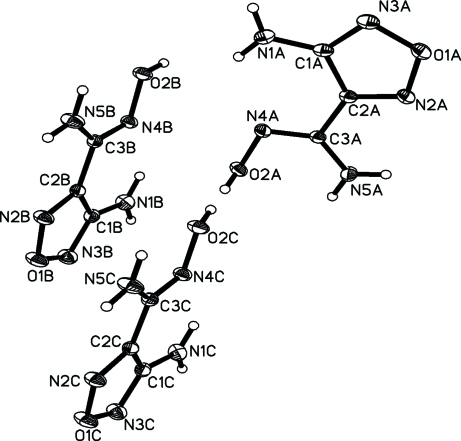
The asymmetric unit of (I), drawn with 30% probability ellipsoids and spheres of arbritrary size for the H atoms.

## supplementary crystallographic information

### Comment

Furazanes (1,2,5-oxadiazoles )have been reported to exhibit a wide spectrum of biological properties (Renaud & Sebastian, 2003). In particular, agrochemical applications of furazanes and their derivatives such as, herbicides, plant-growth regulators and pesticides have been described. As part of our search for new non-linear optically active compounds we synthesized the title compound (I), and report its crystal structure herein. The asymmetric unit of (I) contains three crystallograpically independent molecules (see Fig. 1). In the crystal structure intermolecular N-H···N, N-H···O, O-H···N and O-H···O hydrogen bonds link molecules into a three-dimensional network.

### Experimental

A mixture of propanedinitrile (0.1mol) hydroxylamine hydrochloride (0.22 mol) and 40% NaOH (10ml PH=10) was stirred in water (200mL) for 10h [diazo-reaction] to afford the title compound (yield 43%). Single crystals suitable for X-ray measurements were obtained by recrystallization from  water and ethanol at room temperature.

### Refinement

The H atoms were placed in calculated positions (N-H = 0.86; O-H = 0.82Å) and refined in a riding-motion approximation with U_iso_(H) = 1.2U_eq_(N,O).

### Crystal data

**Table:**

C_3_H_5_N_5_O_2_	*F*(000) = 888
*M**_r_* = 143.12	*D*_x_ = 1.667 Mg m^−^^3^
Monoclinic, *P*2_1_/*c*	Mo *K*α radiation, λ = 0.71073 Å
Hall symbol: -P 2ybc	Cell parameters from 3891 reflections
*a* = 7.6514 (15) Å	θ = 3.2–27.5°
*b* = 11.712 (2) Å	µ = 0.14 mm^−^^1^
*c* = 19.218 (4) Å	*T* = 293 K
β = 96.53 (3)°	Bar, yellow
*V* = 1710.9 (6) Å^3^	0.20 × 0.15 × 0.10 mm
*Z* = 12	

### Data collection

**Table:**

Bruker SMART CCD diffractometer	2954 reflections with *I* > 2σ(*I*)
Radiation source: fine-focus sealed tube	*R*_int_ = 0.018
graphite	θ_max_ = 27.5°, θ_min_ = 3.2°
φ and ω scans	*h* = −8→9
16421 measured reflections	*k* = −15→15
3891 independent reflections	*l* = −24→24

### Refinement

**Table:**

Refinement on *F*^2^	Primary atom site location: structure-invariant direct methods
Least-squares matrix: full	Secondary atom site location: difference Fourier map
*R*[*F*^2^ > 2σ(*F*^2^)] = 0.041	Hydrogen site location: inferred from neighbouring sites
*wR*(*F*^2^) = 0.131	H-atom parameters constrained
*S* = 0.88	*w* = 1/[σ^2^(*F*_o_^2^) + (0.0979*P*)^2^ + 0.2809*P*] where *P* = (*F*_o_^2^ + 2*F*_c_^2^)/3
3891 reflections	(Δ/σ)_max_ < 0.001
271 parameters	Δρ_max_ = 0.41 e Å^−^^3^
0 restraints	Δρ_min_ = −0.27 e Å^−^^3^

### Special details

**Table:**

Geometry. All e.s.d.'s (except the e.s.d. in the dihedral angle between two l.s. planes) are estimated using the full covariance matrix. The cell e.s.d.'s are taken into account individually in the estimation of e.s.d.'s in distances, angles and torsion angles; correlations between e.s.d.'s in cell parameters are only used when they are defined by crystal symmetry. An approximate (isotropic) treatment of cell e.s.d.'s is used for estimating e.s.d.'s involving l.s. planes.
Refinement. Refinement of *F*^2^ against ALL reflections. The weighted *R*-factor *wR* and goodness of fit *S* are based on *F*^2^, conventional *R*-factors *R* are based on *F*, with *F* set to zero for negative *F*^2^. The threshold expression of *F*^2^ > σ(*F*^2^) is used only for calculating *R*-factors(gt) *etc*. and is not relevant to the choice of reflections for refinement. *R*-factors based on *F*^2^ are statistically about twice as large as those based on *F*, and *R*- factors based on ALL data will be even larger.

### Fractional atomic coordinates and isotropic or equivalent isotropic displacement parameters (Å^2^)

**Table:**

	*x*	*y*	*z*	*U*_iso_*/*U*_eq_	
O2C	0.33315 (11)	0.21704 (8)	0.19898 (5)	0.0347 (2)	
H2CA	0.3466	0.2671	0.2289	0.042*	
N4C	0.50875 (13)	0.20169 (9)	0.18228 (5)	0.0286 (2)	
O1C	0.87029 (12)	0.05568 (9)	0.03919 (5)	0.0401 (3)	
N2C	0.69431 (13)	0.05829 (9)	0.04686 (6)	0.0329 (3)	
C3C	0.50579 (14)	0.14024 (9)	0.12631 (6)	0.0246 (3)	
C2C	0.67858 (15)	0.11797 (9)	0.10272 (6)	0.0245 (3)	
N5C	0.36404 (14)	0.09580 (10)	0.08818 (6)	0.0388 (3)	
H5CA	0.2608	0.1073	0.1004	0.047*	
H5CB	0.3765	0.0558	0.0515	0.047*	
N1C	0.89742 (14)	0.21284 (9)	0.19346 (5)	0.0333 (3)	
H1CA	0.9980	0.2464	0.1975	0.040*	
H1CB	0.8122	0.2534	0.2056	0.040*	
C1C	0.85080 (14)	0.15425 (10)	0.13211 (6)	0.0261 (3)	
N3C	0.96673 (14)	0.11600 (10)	0.09296 (6)	0.0355 (3)	
O2A	0.32799 (11)	0.39453 (7)	0.29805 (4)	0.0308 (2)	
H2	0.3073	0.4390	0.2652	0.037*	
N4A	0.15351 (12)	0.37447 (9)	0.31574 (5)	0.0275 (2)	
C3A	0.15895 (14)	0.31171 (9)	0.37121 (6)	0.0230 (2)	
O1A	−0.21344 (12)	0.22039 (9)	0.45154 (5)	0.0401 (3)	
N5A	0.30087 (14)	0.26718 (10)	0.40878 (6)	0.0380 (3)	
H5AA	0.4039	0.2795	0.3966	0.046*	
H5AB	0.2889	0.2263	0.4451	0.046*	
N2A	−0.03621 (13)	0.22429 (9)	0.44691 (6)	0.0318 (3)	
N1A	−0.22143 (14)	0.38907 (10)	0.30266 (6)	0.0376 (3)	
H1AA	−0.3287	0.4043	0.2870	0.045*	
H1AB	−0.1370	0.4140	0.2809	0.045*	
C2A	−0.01584 (14)	0.28737 (10)	0.39297 (6)	0.0249 (3)	
C1A	−0.18545 (15)	0.32568 (10)	0.36160 (6)	0.0279 (3)	
N3A	−0.30561 (14)	0.28438 (10)	0.39787 (6)	0.0377 (3)	
O2B	−0.00634 (11)	0.55083 (8)	0.19706 (5)	0.0322 (2)	
H2BA	0.0138	0.5963	0.2294	0.039*	
O1B	0.52696 (12)	0.37960 (9)	0.03821 (5)	0.0398 (3)	
N4B	0.16666 (12)	0.52895 (9)	0.17977 (5)	0.0268 (2)	
C3B	0.16061 (14)	0.47025 (9)	0.12245 (6)	0.0235 (2)	
C2B	0.33383 (14)	0.44556 (9)	0.09947 (6)	0.0236 (2)	
C1B	0.50497 (14)	0.48252 (10)	0.12947 (6)	0.0260 (3)	
N1B	0.54677 (14)	0.54437 (10)	0.18905 (6)	0.0346 (3)	
H1BA	0.6551	0.5585	0.2035	0.042*	
H1BB	0.4648	0.5693	0.2122	0.042*	
N5B	0.01713 (13)	0.43080 (10)	0.08336 (6)	0.0363 (3)	
H5BA	−0.0856	0.4439	0.0957	0.044*	
H5BB	0.0277	0.3924	0.0459	0.044*	
N2B	0.35054 (13)	0.38407 (9)	0.04455 (6)	0.0328 (3)	
N3B	0.62218 (14)	0.44196 (10)	0.09165 (6)	0.0353 (3)	

### Atomic displacement parameters (Å^2^)

**Table:**

	*U*^11^	*U*^22^	*U*^33^	*U*^12^	*U*^13^	*U*^23^
O2C	0.0275 (5)	0.0428 (5)	0.0354 (5)	0.0058 (4)	0.0103 (4)	−0.0068 (4)
N4C	0.0221 (5)	0.0355 (5)	0.0290 (5)	0.0019 (4)	0.0066 (4)	−0.0024 (4)
O1C	0.0256 (5)	0.0562 (6)	0.0402 (5)	0.0016 (4)	0.0112 (4)	−0.0122 (4)
N2C	0.0227 (5)	0.0427 (6)	0.0341 (6)	−0.0002 (4)	0.0074 (4)	−0.0072 (5)
C3C	0.0202 (5)	0.0271 (5)	0.0272 (6)	0.0008 (4)	0.0053 (4)	0.0021 (4)
C2C	0.0209 (6)	0.0276 (5)	0.0253 (6)	0.0005 (4)	0.0036 (4)	0.0020 (4)
N5C	0.0209 (5)	0.0532 (7)	0.0430 (7)	−0.0033 (5)	0.0069 (5)	−0.0193 (5)
N1C	0.0265 (5)	0.0407 (6)	0.0323 (6)	−0.0065 (4)	0.0016 (4)	−0.0011 (4)
C1C	0.0203 (6)	0.0308 (6)	0.0275 (6)	−0.0001 (4)	0.0039 (4)	0.0056 (4)
N3C	0.0244 (5)	0.0470 (6)	0.0358 (6)	−0.0007 (4)	0.0064 (4)	−0.0026 (5)
O2A	0.0260 (4)	0.0395 (5)	0.0282 (5)	−0.0026 (3)	0.0086 (3)	0.0051 (4)
N4A	0.0202 (5)	0.0359 (5)	0.0270 (5)	−0.0001 (4)	0.0048 (4)	0.0034 (4)
C3A	0.0192 (5)	0.0264 (5)	0.0237 (6)	0.0007 (4)	0.0039 (4)	−0.0013 (4)
O1A	0.0249 (5)	0.0514 (6)	0.0460 (6)	−0.0017 (4)	0.0132 (4)	0.0107 (4)
N5A	0.0189 (5)	0.0557 (7)	0.0397 (6)	0.0051 (5)	0.0049 (4)	0.0211 (5)
N2A	0.0225 (5)	0.0391 (6)	0.0349 (6)	−0.0005 (4)	0.0083 (4)	0.0059 (4)
N1A	0.0246 (5)	0.0492 (7)	0.0382 (6)	0.0073 (5)	−0.0004 (4)	0.0059 (5)
C2A	0.0198 (6)	0.0286 (5)	0.0263 (6)	0.0009 (4)	0.0037 (4)	−0.0019 (4)
C1A	0.0193 (6)	0.0321 (6)	0.0321 (6)	0.0009 (4)	0.0026 (5)	−0.0054 (5)
N3A	0.0216 (5)	0.0469 (6)	0.0451 (7)	0.0012 (5)	0.0066 (5)	0.0017 (5)
O2B	0.0227 (4)	0.0439 (5)	0.0307 (5)	0.0038 (4)	0.0067 (3)	−0.0051 (4)
O1B	0.0241 (5)	0.0566 (6)	0.0404 (5)	0.0014 (4)	0.0108 (4)	−0.0122 (4)
N4B	0.0195 (5)	0.0359 (5)	0.0254 (5)	0.0014 (4)	0.0050 (4)	−0.0011 (4)
C3B	0.0192 (5)	0.0274 (5)	0.0241 (6)	−0.0001 (4)	0.0033 (4)	0.0034 (4)
C2B	0.0204 (6)	0.0274 (5)	0.0233 (5)	−0.0003 (4)	0.0032 (4)	0.0021 (4)
C1B	0.0187 (5)	0.0305 (6)	0.0287 (6)	−0.0013 (4)	0.0028 (4)	0.0050 (4)
N1B	0.0258 (5)	0.0437 (6)	0.0332 (6)	−0.0051 (4)	−0.0012 (4)	−0.0059 (5)
N5B	0.0189 (5)	0.0530 (7)	0.0371 (6)	−0.0025 (5)	0.0036 (4)	−0.0158 (5)
N2B	0.0218 (5)	0.0444 (6)	0.0331 (6)	−0.0007 (4)	0.0072 (4)	−0.0072 (5)
N3B	0.0231 (5)	0.0469 (6)	0.0362 (6)	−0.0013 (4)	0.0053 (4)	−0.0031 (5)

### Geometric parameters (Å, °)

**Table:**

O2C—N4C	1.4278 (12)	N5A—H5AB	0.8600
O2C—H2CA	0.8200	N2A—C2A	1.2966 (15)
N4C—C3C	1.2922 (15)	N1A—C1A	1.3560 (16)
O1C—N2C	1.3715 (13)	N1A—H1AA	0.8600
O1C—N3C	1.3921 (15)	N1A—H1AB	0.8600
N2C—C2C	1.2979 (16)	C2A—C1A	1.4391 (16)
C3C—N5C	1.3429 (15)	C1A—N3A	1.3080 (16)
C3C—C2C	1.4690 (16)	O2B—N4B	1.4242 (12)
C2C—C1C	1.4375 (16)	O2B—H2BA	0.8199
N5C—H5CA	0.8600	O1B—N2B	1.3701 (13)
N5C—H5CB	0.8600	O1B—N3B	1.3959 (15)
N1C—C1C	1.3757 (16)	N4B—C3B	1.2949 (15)
N1C—H1CA	0.8600	C3B—N5B	1.3399 (15)
N1C—H1CB	0.8600	C3B—C2B	1.4725 (15)
C1C—N3C	1.3059 (16)	C2B—N2B	1.2959 (15)
O2A—N4A	1.4339 (12)	C2B—C1B	1.4361 (15)
O2A—H2	0.8199	C1B—N3B	1.3063 (15)
N4A—C3A	1.2916 (15)	C1B—N1B	1.3620 (16)
C3A—N5A	1.3394 (15)	N1B—H1BA	0.8600
C3A—C2A	1.4738 (15)	N1B—H1BB	0.8600
O1A—N2A	1.3698 (13)	N5B—H5BA	0.8600
O1A—N3A	1.3990 (15)	N5B—H5BB	0.8600
N5A—H5AA	0.8600		
			
N4C—O2C—H2CA	101.7	C1A—N1A—H1AA	120.0
C3C—N4C—O2C	109.23 (10)	C1A—N1A—H1AB	120.0
N2C—O1C—N3C	110.66 (9)	H1AA—N1A—H1AB	120.0
C2C—N2C—O1C	106.59 (10)	N2A—C2A—C1A	109.14 (10)
N4C—C3C—N5C	127.35 (11)	N2A—C2A—C3A	122.18 (10)
N4C—C3C—C2C	115.13 (10)	C1A—C2A—C3A	128.68 (11)
N5C—C3C—C2C	117.52 (11)	N3A—C1A—N1A	124.01 (11)
N2C—C2C—C1C	108.48 (10)	N3A—C1A—C2A	108.45 (11)
N2C—C2C—C3C	121.49 (11)	N1A—C1A—C2A	127.47 (11)
C1C—C2C—C3C	130.03 (11)	C1A—N3A—O1A	105.37 (10)
C3C—N5C—H5CA	120.0	N4B—O2B—H2BA	101.2
C3C—N5C—H5CB	120.0	N2B—O1B—N3B	110.58 (9)
H5CA—N5C—H5CB	120.0	C3B—N4B—O2B	110.48 (9)
C1C—N1C—H1CA	116.6	N4B—C3B—N5B	127.43 (11)
C1C—N1C—H1CB	112.2	N4B—C3B—C2B	114.40 (10)
H1CA—N1C—H1CB	114.9	N5B—C3B—C2B	118.18 (10)
N3C—C1C—N1C	122.54 (11)	N2B—C2B—C1B	108.93 (10)
N3C—C1C—C2C	108.95 (11)	N2B—C2B—C3B	121.93 (10)
N1C—C1C—C2C	128.38 (11)	C1B—C2B—C3B	129.13 (11)
C1C—N3C—O1C	105.32 (10)	N3B—C1B—N1B	123.41 (11)
N4A—O2A—H2	100.6	N3B—C1B—C2B	108.63 (11)
C3A—N4A—O2A	110.21 (9)	N1B—C1B—C2B	127.88 (11)
N4A—C3A—N5A	127.92 (11)	C1B—N1B—H1BA	120.0
N4A—C3A—C2A	113.53 (10)	C1B—N1B—H1BB	120.0
N5A—C3A—C2A	118.55 (10)	H1BA—N1B—H1BB	120.0
N2A—O1A—N3A	110.73 (9)	C3B—N5B—H5BA	120.0
C3A—N5A—H5AA	120.0	C3B—N5B—H5BB	120.0
C3A—N5A—H5AB	120.0	H5BA—N5B—H5BB	120.0
H5AA—N5A—H5AB	120.0	C2B—N2B—O1B	106.45 (10)
C2A—N2A—O1A	106.31 (10)	C1B—N3B—O1B	105.40 (10)

### Hydrogen-bond geometry (Å, °)

**Table:**

*D*—H···*A*	*D*—H	H···*A*	*D*···*A*	*D*—H···*A*
O2C—H2CA···O2A	0.82	2.01	2.8222 (14)	169
O2A—H2···N4B	0.82	2.14	2.9208 (14)	160
N5C—H5CA···N3C^i^	0.86	2.24	3.0610 (17)	160
N5C—H5CB···N2C^ii^	0.86	2.33	3.1508 (17)	160
N1C—H1CA···O2C^iii^	0.86	2.58	3.3239 (15)	145
N1C—H1CB···N1A^iii^	0.86	2.49	3.1504 (17)	135
N1C—H1CB···N4C	0.86	2.39	2.9600 (16)	124
N5A—H5AA···N3A^iii^	0.86	2.22	3.0486 (17)	162
N5A—H5AB···N2B^iv^	0.86	2.31	3.1409 (17)	162
N1A—H1AA···N1B^i^	0.86	2.60	3.2156 (17)	130
N1A—H1AB···O2B	0.86	2.56	3.3430 (17)	153
N1A—H1AB···N4A	0.86	2.29	2.8563 (15)	123
O2B—H2BA···N1C^v^	0.82	2.07	2.8849 (15)	171
N1B—H1BB···N4B	0.86	2.34	2.8992 (15)	123
N1B—H1BB···N4C^v^	0.86	2.54	3.1510 (17)	129
N5B—H5BA···N3B^i^	0.86	2.23	3.0470 (16)	159
N5B—H5BB···N2A^vi^	0.86	2.35	3.1779 (17)	162

Symmetry codes: (i) *x*−1, *y*, *z*; (ii) −*x*+1, −*y*, −*z*; (iii) *x*+1, *y*, *z*; (iv) *x*, −*y*+1/2, *z*+1/2; (v) −*x*+1, *y*+1/2, −*z*+1/2; (vi) *x*, −*y*+1/2, *z*−1/2.

## References

[bb1] Bruker (1997). *SMART* and *SAINT* Bruker AXS Inc., Madison, Wisconsin, USA.

[bb2] Renaud, B. & Sebastian, W. (2003). *Heterocycles*, **60**, 2417–2424.

[bb3] Sheldrick, G. M. (2008). *Acta Cryst.* A**64**, 112–122. 10.1107/S0108767307043930 18156677

